# Belantamab mafodotin, pomalidomide, and dexamethasone in Japanese patients with RRMM in the phase 3 DREAMM-8 trial

**DOI:** 10.1007/s12185-025-04150-6

**Published:** 2026-01-06

**Authors:** Kazutaka Sunami, Hiroshi Handa, Michiko Ichii, Takayuki Ikezoe, Kazuhito Suzuki, Yusuke Yamaguchi, Taeko Yonekawa, Akira Endo, Hirofumi Nakano, Eric Lewis, Ianire Garrobo Calleja, Elisabet Manasanch, Shigeki Ito, Hitomi Kato

**Affiliations:** 1 Department of Hematology, National Hospital Organization Okayama Medical Center, 1711-1 Tamasu, Kita-ku, Okayama City, Japan; 2 Department of Hematology, Gunma University Graduate School of Medicine, Gunma, Japan; 3 The University of Osaka Graduate School of Medicine, Osaka, Japan; 4 Fukushima Medical University, Fukushima, Japan; 5 The Jikei University School of Medicine, Tokyo, Japan; 6 GSK Tokyo, 1-8-1, Akasaka, Minato-ku, Tokyo, Japan; 7 GSK, Durham, NC USA; 8 GSK, London, UK; 9 GSK, Philadelphia, PA USA; 10 Division of Hematology & Oncology, Iwate Medical University School of Medicine, Iwate, Japan

**Keywords:** B-cell maturation antigen, Belantamab mafodotin, Japan, Relapsed/refractory multiple myeloma

## Abstract

The phase 3 DREAMM-8 (NCT04484623) trial assessed belantamab mafodotin + pomalidomide + dexamethasone (BPd) vs bortezomib + Pd (PVd) in lenalidomide-exposed patients with relapsed/refractory multiple myeloma (RRMM) and ≥ 1 prior therapy. Here, we present findings from Japanese patients (data cutoff: 27-May-24). The primary endpoint was progression-free survival (PFS). Secondary endpoints included overall response rate (ORR), duration of response (DoR), and safety. Overall, 21 patients were randomized 1:1 to BPd (N = 10) and PVd (N = 11). Median follow-up was 13.8 months (range 0.23–26.71). For BPd vs PVd, median PFS was not reached (NR; 95% CI 0.2–NR) vs 14.8 months (95% CI 1.9–NR; HR 0.53; 95% CI 0.1–2.8); ORR was 90% (9/10) vs 73% (8/11), including very good partial response or better in 70% (7/10) vs 36% (4/11); median DoR was NR vs 17.5 months. The safety profile was consistent with the global cohort. Ocular adverse events were more common with BPd vs PVd (90% [9/10] vs 9% [1/11]) and a majority were transient and reversible. While the sample size of this study is small, findings were aligned with the global cohort. BPd showed favorable efficacy compared with PVd, with a manageable safety profile in lenalidomide-exposed Japanese patients with RRMM.

**Supplementary Information:**

The online version contains supplementary material available at 10.1007/s12185-025-04150-6.

## Introduction

The standard of care for patients with newly diagnosed multiple myeloma (MM) includes treatment with triplet or quadruplet regimens that combine proteasome inhibitors, immunomodulators, and anti-CD38 monoclonal antibodies [1–3]. Despite the increasing number of available treatment options, most patients with MM frequently relapse or become refractory to treatment.

Treatment options for relapsed and/or refractory MM (RRMM) depend on several factors, including the patient’s prior treatment history, molecular and genetic characteristics of the disease, overall health status, and any comorbid conditions [4]. Based on recent treatment guidelines, lenalidomide and anti-CD38 monoclonal antibodies (such as daratumumab) are increasingly used globally as first-line treatment regimens [2, 5–7]. However, the widespread use of these agents in the frontline setting can limit their options for patients with RRMM. Emerging data have shown that patients who are refractory to lenalidomide are associated with suboptimal progression-free survival (PFS) and overall survival (OS) outcomes in subsequent lines of treatment [8]. Furthermore, retreatment with anti-CD38-based therapies in patients with RRMM has shown variable clinical outcomes and limited clinical benefit [9]. Overall, this highlights the need for novel treatments that target different mechanisms of action for patients with RRMM.

In patients with MM, B-cell maturation antigen (BCMA), a member of the tumor necrosis factor receptor superfamily, is overexpressed on malignant plasma cells, which makes it a good target for therapeutic antibodies with cytotoxic activity [10]. Belantamab mafodotin is a humanized, afucosylated anti-BCMA monoclonal antibody conjugated to the microtubule inhibitor monomethyl auristatin-F by a protease-resistant cysteine linker [11, 12]. It has demonstrated promising efficacy in clinical trials, leading to its recent approval in combination regimens for RRMM in Japan, the UK, and the European Union [13–18]. The global phase 3 DREAMM-8 trial (NCT04484623) evaluated belantamab mafodotin + pomalidomide + dexamethasone (BPd) vs pomalidomide + bortezomib + dexamethasone (PVd) in patients with RRMM with ≥ 1 prior therapy, including lenalidomide [16]. The study demonstrated that BPd was associated with a statistically significant and clinically meaningful reduction in the risk of disease progression and death compared with PVd. Although the incidence of ocular adverse events (AEs) was greater with BPd than PVd, all events were manageable with dose modifications and were generally reversible. Here, we present an analysis of Japanese patients enrolled in the DREAMM-8 trial, including patients from the global cohort and the Japan expansion cohort.

## Methods

### Patients and study design

The methodology of the DREAMM-8 study has been previously reported [16]. In brief, DREAMM-8 is an ongoing, open-label, global, phase 3, randomized trial assessing the efficacy and safety of BPd and PVd in patients with RRMM who had been treated with at least one prior line of therapy, including lenalidomide. This analysis of the Japanese population included ethnically Japanese patients who were treated in Japan from the global cohort and the Japan expansion cohort; the Japan expansion cohort enabled Japanese patients to be enrolled into the study after completion of the global cohort recruitment.

Eligible patients were ≥ 18 years of age with a confirmed MM diagnosis, an Eastern Cooperative Oncology Group (ECOG) performance status of 0–2, and previously treated with ≥ 1 prior line of MM therapy, including a lenalidomide-based regimen, who had documented disease progression during or after their most recent therapy. A full list of inclusion and exclusion criteria is presented in the Supplementary Materials. Patients were excluded if they received prior BCMA-targeted therapy, received or were intolerant to pomalidomide, or were intolerant or refractory to bortezomib at 1.3 mg/m^2^ twice weekly. Patients were stratified based on the number of prior lines of therapy (1 vs 2 or 3 vs ≥ 4), and prior bortezomib treatment (yes or no) and prior anti-CD38 treatment (yes or no).

Patients were randomized 1:1 to receive either BPd or PVd (Fig. S1). Patients in the BPd group received 28-day cycles of belantamab mafodotin (2.5 mg/kg of body weight intravenously on day 1 of cycle 1 and 1.9 mg/kg on day 1 of cycle 2 onward) combined with pomalidomide (4 mg per day orally on days 1–21 of each 28-day cycle) and dexamethasone (40 mg per day orally on days 1, 8, 15, and 22 of each 28-day cycle). Patients > 75 years of age, those who had comorbidities, or those who were intolerant to dexamethasone 40 mg, were administered dexamethasone at 20 mg per day at the discretion of the investigator. Patients in the PVd group received 21-day cycles of bortezomib (1.3 mg per square meter of body-surface area subcutaneously on days 1, 4, 8, and 11 of cycles 1 through 8 and days 1 and 8 of cycle 9 onward) combined with pomalidomide (4 mg per day orally on days 1–14 of each 21-day cycle), and dexamethasone (20 mg orally on the day of and the day after bortezomib of each 21-day cycle or on days 1, 2, 4, 5, 8, 9, 11, and 12 of each 21-day cycle for cycles 1 through 8 and then on days 1, 2, 8, and 9 of each 21-day cycle for cycle 9 onwards). Treatment continued until progressive disease, death, unacceptable toxicities, start of new anti-myeloma therapy, patient withdrawal of consent, or end of the study, whichever occurred first. Dose delays and reductions were permitted following potential drug-associated toxicities. To determine whether dose modification was required, patients receiving BPd underwent ophthalmic examination prior to each dose of belantamab mafodotin, up to at least the sixth dose. Dose modifications for belantamab mafodotin treatment-related corneal events were based on Keratopathy Visual Acuity (KVA) scale. For grade 1 KVA events, treatment continued at the current dose. For grade 2 or grade 3 KVA events, treatment was withheld until recovery to grade ≤ 1. If toxicity was identified prior to dosing cycle 2, the patient should restart at 1.9 mg/kg every 4 weeks, as planned. If toxicity was identified after cycle 2, the dosing interval should be extended to 8 weeks. For grade 4 events, treatment was withheld until recovery to grade ≤ 1 and potentially restarted at 1.4 mg/kg every 8 weeks following a benefit/risk assessment. Further information on dose modification guidelines for belantamab mafodotin treatment-related AEs and corneal-related AEs are included in Table S1.

The trial was sponsored by GSK and conducted following the Declaration of Helsinki and Good Clinical Practice guidelines. GSK representatives participated in the data collection, analysis, and interpretation. All patients provided written informed consent prior to enrollment. The trial protocol and its amendments received approval from the relevant ethics committee at each participating institution.

### Endpoints and assessments

The primary endpoint was PFS, based on assessment by an independent review committee (IRC). Key secondary endpoints included OS, minimal residual disease (MRD)–negative status, and duration of response (DoR). All patients in both groups underwent response assessments every 4 weeks. Additional secondary endpoints included overall response rate (ORR; defined as partial response or better) and AEs, which were graded in accordance with the Common Terminology Criteria for Adverse Events (CTCAE), version 5.0. AEs of special interest (AESI) for belantamab mafodotin included ocular AEs, thrombocytopenia, and infusion-related reactions. In addition, corneal events were graded with the KVA scale, which incorporates findings on corneal examination and changes in the best corrected visual acuity (BCVA) to generate a composite grade.

### Statistical analysis

The efficacy analyses were based on intention-to-treat. The distribution of PFS was estimated using the Kaplan–Meier method, with confidence intervals (CI) for the median, and the first and third quartiles estimated using the Brookmeyer Crowley method. The treatment effect (hazard ratio [HR]) with its 95% CI was estimated using an unstratified Cox proportional hazard model with only the treatment arm as the explanatory variable. The distributions of OS and DoR were also estimated using the Kaplan–Meier method, with CIs for median, and the first and third quartiles estimated using the Brookmeyer Crowley method. For ORR based on IRC assessment per International Myeloma Working Group as the best overall response and MRD negativity rate, the corresponding exact 95% CI was provided for each treatment arm. The safety analyses were based on the safety population, defined as all randomized patients who took at least one dose of study treatment. This Japanese population analysis was conducted after the statistically significant and clinically meaningful improvement in PFS was demonstrated in the global cohort [16].

## Results

### Patients and treatment

From January 2022 to February 2024, a total of 21 Japanese patients were enrolled: 9 from the global cohort and 12 from the Japan expansion cohort. Overall, 10 patients were randomized to BPd and 11 patients to PVd. At data cutoff, 60% of patients (6/10) in the BPd group and 27% of patients (3/11) in the PVd group continued study treatment, 10% (1/10) in the BPd group and 64% (7/11) in the PVd group were in follow-up, and 20% (2/10) in the BPd group and 9% (1/11) in the PVd group died. One patient in the BPd group withdrew from the study. The median duration of follow-up was 12.9 months (range 0.2–26.6) in the BPd group and 14.5 months (range 6.0–26.7) in the PVd group, with a minimum ongoing follow-up of 2.8 and 6.0 months, respectively.

Baseline patient characteristics and previous treatments received were generally similar in the two groups, except for International Staging System (ISS) stage III (BPd 20% [2/10] and PVd 0%), extramedullary disease (BPd 20% [2/10] and PVd 0%), and high-risk cytogenetics (at least one high-risk abnormality: t(4;14), t(14;16), or 17p13del; BPd 30% [3/10] and PVd 9% [1/11]) (Table 1). More patients in the BPd group were refractory to lenalidomide (BPd 80% [8/10] and PVd 36% [4/11]), anti-CD38 antibodies (BPd 40% [4/10] and PVd 27% [3/11]), and proteasome inhibitors (BPd 40% [4/10] and PVd 9% [1/11]) than the PVd group. The median total duration of treatment exposure was 6.8 months (range 0.9–26.7) in the BPd group and 6.2 months (range 0.7–26.9) in the PVd group. The BPd group received a median of 7 treatment cycles (range 1–29) of any study treatment while PVd received median of 8 treatment cycles (range 1–35). The overall median relative dose intensity was 76.3% for belantamab mafodotin in the BPd group and 98.2% for bortezomib in the PVd group.

**Table 1:** Baseline demographic and clinical characteristics and previous therapies

	BPd (N = 10)	PVd (N = 11)	Total (N = 21)
Age, mean (SD), years	72.6 (6.0)	72.5 (5.7)	72.6 (5.7)
Race, n (%)			
Asian (Japanese heritage)	10 (100)	11 (100)	21 (100)
ECOG performance status^a^, n (%)			
0	9 (90)	7 (64)	–
1	0	4 (36)	–
2	1 (10)	0	–
ISS stage at screening, n (%)			
I	5 (50)	6 (55)	11 (52)
II	2 (20)	5 (45)	7 (33)
III	2 (20)	0	2 (10)
Unknown	1 (10)	0	1 (5)
Cytogenetic risk^b^, n (%)			
Standard	7 (70)	9 (82)	16 (76)
High	3 (30)	1 (9)	4 (19)
t(4;14)	2 (20)	0	2 (10)
t(14;16)	0	0	0
del(17p13)	1 (10)	1 (9)	2 (10)
Missing or not evaluable	0	1 (9)	1 (5)
Extramedullary disease, n (%)			
No	8 (80)	11 (100)	19 (90)
Yes	2 (20)	0	2 (10)
Myeloma IgG, n (%)	6 (60)	6 (55)	12 (57)
Lines of therapy completed prior to screening			
Median (range)	1 (1, 8)	1 (1, 3)	1 (1, 8)
Previous lines of therapy, n (%)			
1	7 (70)	8 (73)	15 (71)
2 or 3	2 (20)	3 (27)	5 (24)
≥ 4	1 (10)	0	1 (5)
Prior stem cell transplant, n (%)			
No	6 (60)	8 (73)	14 (67)
Yes	4 (40)	3 (27)	7 (33)
Refractory to prior therapies, n (%)			
Immunomodulator			
Lenalidomide	8 (80)	4 (36)	12 (57)
Steroids	7 (70)	3 (27)	10 (48)
Monoclonal antibody			
Anti-CD38			
Daratumumab	4 (40)	3 (27)	7 (33)
Isatuximab	1 (10)	1 (9)	2 (10)
Other			
Elotuzumab	1 (10)	0	1 (5)
Proteosome inhibitor			
Ixazomib	2 (20)	0	2 (10)
Bortezomib	1 (10)	0	1 (5)
Carfilzomib	1 (10)	1 (9)	2 (10)

*BPd* belantamab mafodotin, pomalidomide, and dexamethasone, *ECOG* Eastern Cooperative Oncology Group, *IgG* immunoglobulin G, *ISS* International Staging System, *PVd* pomalidomide, bortezomib, and dexamethasone, *SD* standard deviation

^a^ECOG performance-status scores range from 0 to 5, with higher scores indicating greater disability

^b^Standard cytogenetic risk was defined by negative results for all high-risk abnormalities: t(4;14), t(14;16), and del(17p13). High cytogenetic risk was defined by the presence of at least one high-risk abnormality. High-risk cytogenetics was assessed by fluorescence in situ hybridization

### Efficacy

**Fig. 1:**
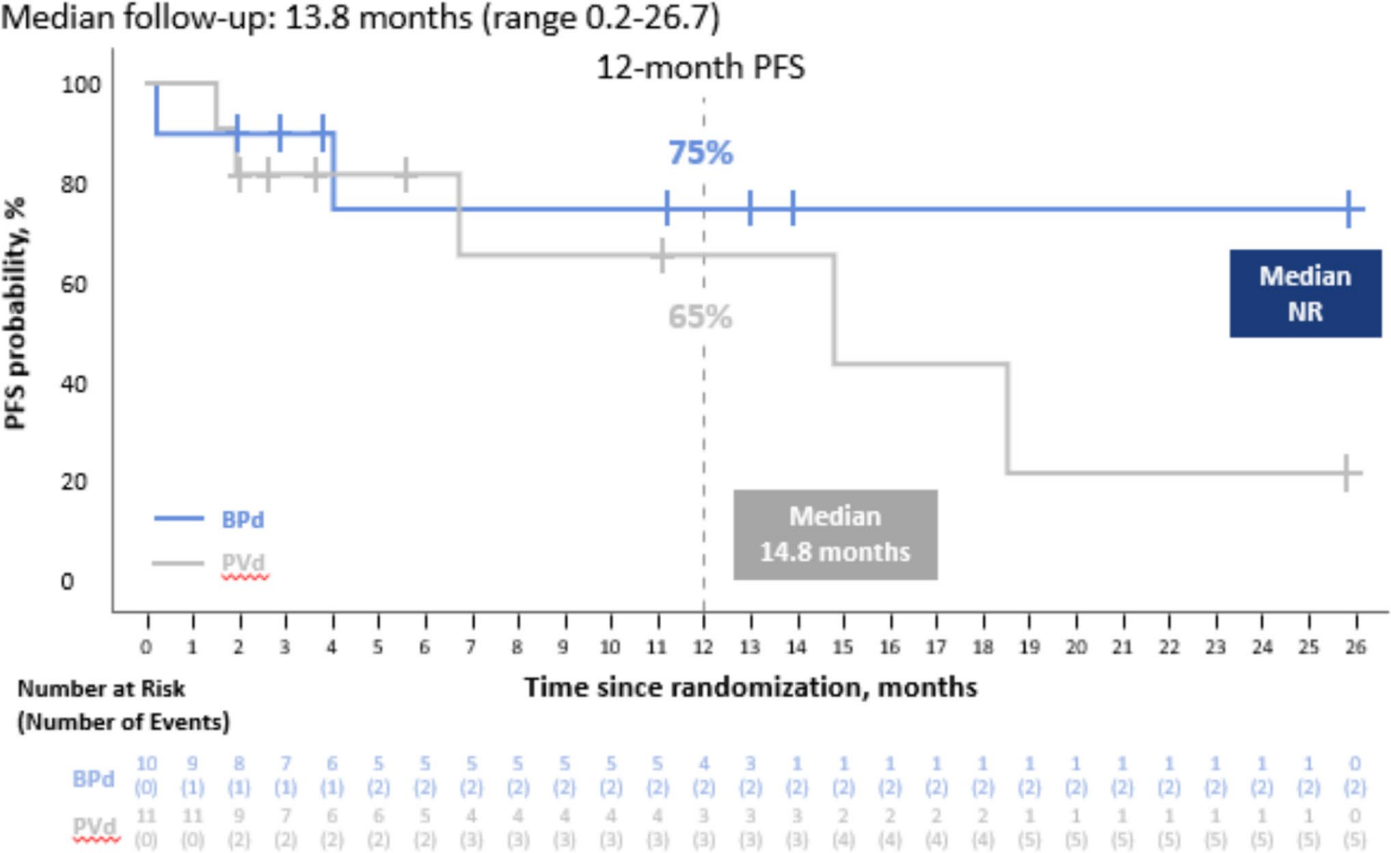
Kaplan–Meier Analysis of Progression-free Survival. BPd, belantamab mafodotin, pomalidomide, and dexamethasone; PFS, progression-free survival; PVd, pomalidomide, bortezomib, and dexamethasone; NR, not reached

The median PFS was not reached (NR; 95% CI, 0.2–NR) in the BPd group and was 14.8 months (95% CI, 1.9–NR) in the PVd group (Fig. 1). The risk of disease progression or death was lower in the BPd group than the PVd group, although the CI for the HR was wide due to the small sample size (HR 0.53; 95% CI, 0.10–2.78). Disease progression or death occurred in 20% (2/10) in the BPd group and 45% (5/11) in the PVd group. In the BPd group, one patient died from fatal sepsis, which was reported as not related to the study treatment. The sepsis was caused by an infection with *Morganella morganii* that started prior to day 1 of study treatment. This was felt to be possibly related to high-dose cytotoxic chemotherapy that the patient had received in the month preceding the start of the study treatment. One patient in each of the BPd and PVd groups died due to cancer. Follow-up for OS is currently ongoing.

The proportion of patients who had an ORR (partial response or better) was 90% (9/10; 95% CI, 55.5–99.7) in the BPd group and 73% (8/11; 95% CI, 39.0–94.0) in the PVd group (Table 2 and Fig. S2). The corresponding proportions of patients with a complete response or better were 30% (3/10; 95% CI 6.7–65.2) and 27% (3/11; 95% CI 6.0–61.0), and a very good partial response or better were 70% (7/10, 95% CI, 34.8–93.3) and 36% (4/11; 95% CI, 10.9–69.2) (Table 2). The proportion of patients with a complete response or better and achieved MRD-negative status was 20% (2/10) in the BPd group and 18% (2/11) in the PVd group (Table S2). Among patients with a partial response or better, the median DoR was NR (95% CI, 1.2–NR) in the BPd group and 17.5 months (95% CI, 13.8–NR) in the PVd group. In the BPd group, 78% (7/9) of patients with response had not progressed or died and had ongoing follow-up for PFS at the data cutoff date, compared with 38% (3/8) in the PVd group.

**Table 2:** Treatment response to BPd and PVd

	BPd (N = 10)	PVd (N = 11)
Best overall response, n (%)		
Stringent complete response	2 (20)	2 (18)
Complete response	1 (10)	1 (9)
Very good partial response	4 (40)	1 (9)
Partial response	2 (20)	4 (36)
Minimal response	0	1 (9)
Stable disease	0	2 (18)
Progressive disease	0	0
Not evaluable	1 (10)	0
Overall response rate, n (%)^a^	9 (90)	8 (73)
95% CI	56–100	39–94
Complete response or better, n (%)	3 (30)	3 (27)
95% CI	7–65	6–61
Very good partial response or better, n (%)	7 (70)	4 (36)
95% CI	35–93	11–69
Median response duration, months (95% CI)^b^	NR (1.2–NR)	17.5 (13.8–NR)

*BPd* belantamab mafodotin, pomalidomide, and dexamethasone, *CI* confidence interval, *NR* not reached, *PVd* pomalidomide, bortezomib, and dexamethasone

^a^Overall response was defined as a partial response or better

^b^Duration of response was defined as the time from the first documented evidence of a partial response or better to the occurrence of disease progression or death from any cause. Response duration was based on n = 9 from BPd and n = 8 from PVd

### Safety

The safety population included 21 patients (10 in the BPd group and 11 in the PVd group). AEs of any grade were experienced by all patients in the BPd and PVd groups (Table S3). AEs leading to permanent discontinuation of any treatment was higher in the PVd group (36%, 4/11) compared to the BPd group (10%, 1/10). The most frequently reported AEs in the BPd group were blurred vision (90%, 9/10), alanine aminotransferase increase (60%, 6/10), constipation (50%, 5/10), aspartate aminotransferase increase (40%, 4/10), diarrhea (40%, 4/10), and foreign body sensation in eyes (40%, 4/10). In the PVd group, the most frequently reported AEs were constipation (64%, 7/11), neutrophil count decrease (45%, 5/11), peripheral edema (36%, 4/11), platelet count decrease (36%, 4/11), thrombocytopenia (36%, 4/11), and white blood cell count decrease (36%, 4/11) (Table 3). Of non-ocular AESIs, the incidence of any grade thrombocytopenia (thrombocytopenia and platelet count decreased) was slightly greater in the PVd group (64%, 7/11) than in the BPd group (60%, 6/10). No infusion-related reactions occurred in the BPd group. The incidence of infections was 70% (7/10) in the BPd group and 36% (4/11) in the PVd group, with grade ≥ 3 infections reported in 40% (4/10) and 18% (2/11), respectively (Table S4). In the BPd group, grade ≥ 3 infections were pneumonia (2 patients), cytomegalovirus chorioretinitis (1 patient), and *Morganella morganii* infection (1 patient). In the PVd group, grade ≥ 3 infections were pneumonia and influenza (1 patient, each).

**Table 3:** Adverse events by preferred term in ≥ 20% of patients in either treatment group (safety population)

	BPd (N = 10)		PVd (N = 11)	
	Any grade	Grade ≥ 3	Any grade	Grade ≥ 3
Any event, n (%)	10 (100)	10 (100)	11 (100)	9 (82)
Blurred vision	9 (90)	2 (20)	1 (9)	0
Alanine aminotransferase increased	6 (60)	1 (10)	2 (18)	1 (9)
Constipation	5 (50)	0	7 (64)	1 (9)
Aspartate aminotransferase increased	4 (40)	1(10)	1 (9)	0
Diarrhea	4 (40)	0	1 (9)	0
Foreign-body sensation in eyes	4 (40)	0	0	0
Neutrophil count decrease	3 (30)	3 (30)	5 (45)	3 (27)
Thrombocytopenia^a^	3 (30)	3 (30)	4 (36)	2 (18)
Cataract	3 (30)	0	2 (18)	1 (9)
Malaise	3 (30)	0	2 (18)	0
Rash	3 (30)	1 (10)	1 (9)	0
Blood alkaline phosphatase increased	3 (30)	1 (10)	0	0
Hypogammaglobulinemia	3 (30)	1 (10)	0	0
Back pain	2 (20)	0	3 (27)	0
Nausea	2 (20)	0	3 (27)	0
Anemia	2 (20)	2 (20)	2 (18)	2 (18)
Dizziness	2 (20)	0	2 (18)	0
Upper respiratory tract infection	2 (20)	0	2 (18)	0
Dry eye	2 (20)	0	1 (9)	0
Hypoesthesia	2 (20)	0	1 (9)	0
Eye irritation	2 (20)	0	0	0
Gamma-glutamyl transferase increased	2 (20)	1 (10)	0	0
Herpes zoster	2 (20)	0	0	0
Vomiting	2 (20)	0	0	0
Oedema peripheral	1 (10)	0	4 (36)	0
White blood cell count decreased	1 (10)	1 (10)	4 (36)	3 (27)
Dysgeusia	1 (10)	0	3 (27)	0
Lymphocyte count decreased	1 (10)	1 (10)	3 (27)	1 (9)
Injection site reaction	0	0	3 (27)	0

*BPd* belantamab mafodotin, pomalidomide, and dexamethasone, *PVd* pomalidomide, bortezomib, and dexamethasone

^a^Adverse event of special interest, including thrombocytopenia and platelet count decreased

At data cutoff, 90% (9/10) of patients in the BPd group experienced CTCAE-graded ocular AEs compared to 9% (1/11) in the PVd group; all ocular AEs were treatment-related (Table 4). The most frequently reported ocular AEs with BPd were blurred vision (90%, 9/10), foreign body sensation in eyes (40%, 4/11), dry eye, and eye irritation (20%, 2/10 each). Grade 3 ocular AEs were reported in two patients in the BPd group, both experiencing blurred vision, and none were reported in the PVd group; no grade 4 ocular AEs were reported. Ocular AEs did not lead to treatment withdrawal or dose reductions in both treatment groups. CTCAE-graded ocular AEs led to dose interruptions or delays in 44% (4/9) of patients who received BPd and none in those who received PVd. The first occurrence of ocular AEs in the BPd group were resolved prior to the end of treatment exposure in 56% (5/9) of patients. Of the four patients with unresolved events, one patient’s event (blurred vision, grade 2 or below) remained unresolved at the end of follow-up (study withdrawal) and was managed without dose modification; the remaining three patients had unresolved events at the data cutoff and remain in follow-up.

**Table 4:** Ocular and corneal events (safety population)

	BPd		PVd	
	Any grade	Grade ≥ 3	Any grade	Grade ≥ 3
Ocular events in ≥ 20% of patients, n (%)^a^	n = 10		n = 11	
Any	9 (90)	2 (20)	1 (9)	0
Blurred vision	9 (90)	2 (20)	1 (9)	0
Foreign-body sensation in eye	4 (40)	0	0	0
Dry eye	2 (20)	0	1 (9)	0
Eye irritation	2 (20)	0	0	0
Action taken for ocular AEs (based on patients with an event), n (%)^b^	n = 9		n = 1	
Study treatment withdrawn	0		0	
Dose reduced	0		0	
Dose not changed	9/9 (100)		1/1 (100)	
Dose interrupted/delayed	4/9 (44)		0	
KVA scale events, n (%)^c^	n = 10		–	
Any event	9 (90)	5 (50)	–	–
Permanent discontinuation of study	0		–	
Dose reduced	5 (50)		–	
Dose interrupted/delayed	8 (80)		–	

*AE* adverse event, *BCVA* Best Corrected Visual Acuity, *BPd* belantamab mafodotin, pomalidomide, and dexamethasone, *CTCAE* Common Terminology Criteria for Adverse Events, *KVA* Keratopathy Visual Acuity, *MedDRA* Medical Dictionary for Regulatory Activities, *PT* preferred term, *PVd* pomalidomide, bortezomib, and dexamethasone, *SMQ* Standardized MedDRA Queries

^a^Ocular AEs (CTCAE grade) were based on the MedDRA ‘corneal disorders’ SMQ combined with PT’s representing changes in visual acuity which were identified by GSK internal review

^b^Patients may have been included in more than one category for ‘Action taken’

^c^Treatment-related corneal events (corneal exam findings and changes in BCVA were graded according to the guidelines of the KVA Scale to inform dose modifications)

Corneal events as defined according to the KVA scale were reported in 90% (9/10) of the patients who received BPd, where 50% (5/10) experienced a KVA grade ≥ 3 event (Table 4). KVA-defined events led to a dose reduction in 50% (5/10) of the patients who received BPd, and a dose interruption or delay in 80% (8/10) of the patients. Of the 8 patients with grade ≥ 2 KVA-defined events, 6 patients had their first event resolved prior to or after the end of treatment exposure and 2 patients remain in follow-up (Fig. S2).

## Discussion

In lenalidomide-exposed Japanese patients with RRMM, BPd demonstrated favorable efficacy compared with PVd, while maintaining a manageable safety profile. This is similar to the primary analysis of the global cohort, which met its primary PFS endpoint, and demonstrated that PFS, ORR, and DoR strongly favored BPd over PVd therapy [16].

In this Japanese cohort, the median PFS was NR for the BPd group, while it was 14.8 months for the PVd group. Notably, Japanese patients who received PVd had a longer median PFS compared to the global cohort (12.7 months) [16] and the OPTIMISMM trial (11.2 months) [19]. Additionally, the DoR for the PVd group was also longer in Japanese patients compared to the global cohort [16] and this is likely due to the high proportion of patients with favorable prognostic factors in the Japanese PVd group. Specifically, in this Japanese cohort, none of the PVd-treated patients had ISS stage III disease. Additionally, compared to the BPd group, fewer PVd-treated patients had extramedullary disease, high-risk cytogenetics, or lenalidomide refractoriness. Furthermore, compared to the global study, this Japanese cohort included more patients with one prior line of therapy for both treatment groups [16]. In this line, a subgroup analysis of patients with one prior line of therapy in the global cohort continued to show a PFS benefit with BPd compared to PVd (NR vs 18.5 months; HR 0.50; 95% CI, 0.30–0.85) [16, 20], which is consistent with the findings in the Japanese cohort (NR vs 14.8 months).

A higher proportion of the BPd group in the Japanese population achieved an objective response compared with the PVd group (90% vs 73%), whereas in the global cohort, ORR was comparable between the treatment arms (77% vs 72%) [16]. Despite the similar ORRs in the global cohort, BPd was associated with a deeper treatment response, with a higher proportion of patients exhibiting a complete response or better (40% vs 16%) and achieving an MRD-negative status (24% vs 5%) compared with PVd [16]. Deeper responses were also observed in this Japanese cohort, whereby the rate of very good partial response or better was greater with BPd compared to PVd (70% vs 36%). In addition, the median DoR was longer in the BPd group compared with the PVd group, for both the Japanese patients (NR vs 17.5 months) and the global cohort (17.5 vs 12.7 months) [16]. However, the DoR for the Japanese population should be interpreted with caution due to the high number of censored data points, which may artificially extend the apparent DoR. OS data was immature in this Japan population, and follow-up for OS is ongoing.

Certain patient or disease characteristics make RRMM more difficult to treat, such as high‐risk cytogenetic abnormalities, renal impairment, the presence of extramedullary disease, old age, and frailty [21]. A recent subgroup analysis of DREAMM-8 demonstrated that compared to PVd, BPd reduced the risk of disease progression or death in patients with high-risk cytogenetics, patients who are refractory to lenalidomide or anti-CD38 treatments, those aged ≥ 75 years, and those with extramedullary disease [22]. In this study, three patients in the BPd group had high-risk cytogenetics, and all three patients achieved a very good partial response or better. Further, 80% of patients in the BPd arm had lenalidomide-refractory disease and 40% had daratumumab-refractory disease. This aligns with recent Japanese real-world data, which showed increasing use of lenalidomide and daratumumab in first-line therapy [7, 23]. With these drugs being more frequently used at first-line, there is a need for effective treatments for patients refractory to lenalidomide and anti-CD38 treatments, as patients with treatment-refractory disease tend to experience suboptimal clinical outcomes [8].

AEs reported in patients who received BPd were consistent with those reported in the global cohort, with no new safety signals [16]. Consistent with the global cohort, the incidence of grade ≥ 3 infections was higher in the BPd group compared with the PVd group [16]. Ocular AEs were common in the BPd group, and often required dose modifications. Nevertheless, none of the ocular AEs resulted in treatment discontinuation in this study. In this analysis of Japanese patients, the most frequently reported ocular AEs in the BPd group were blurred vision and foreign body sensation. Among the patients who had a CTCAE-graded ocular AE, none required a dose reduction but 44% had dose delays. Additionally, among patients who had a KVA-defined corneal event, 56% and 89% of patients had dose reductions and delays respectively. A recent analysis of BPd exposure in the global cohort demonstrated that despite frequent dose modifications to manage ocular AEs, which consequently extended timing between doses, there was no observed impact on efficacy and patients in the BPd group maintained an advantage in ORR and PFS [24]. Further, a post-hoc analysis evaluating the impact of belantamab mafodotin dose modifications in the global cohort reported that ocular events were effectively managed with dose modifications, allowing patients to remain on treatment and derive robust efficacy benefit [25]. This suggests that dose delays are an appropriate approach to manage ocular AEs, while also maintaining clinical benefit. The global cohort also showed that there was no decline in patient-reported quality of life from treatment initiation for either of the treatment arms [16], suggesting that treatment-related ocular AEs did not impact patient quality of life. In the Japan population, the incidence rate for infections was higher in the BPd group than in the PVd group (70% vs 36%), which was also observed in the global cohort (82% vs 68%) [16]. Management of belantamab mafodotin–associated ocular events entail close communication between oncologists and eye care professionals, along with active monitoring of ocular symptoms by patients [26, 27]. Recommended strategies include dose modifications, use of preservative-free lubricant eye drops, and avoiding the use of contact lenses [26]. Implementing standardized ophthalmologic referral pathways and improving patient access to support resources may improve the real-world applicability of BPd [28].

Limitations of this study include its small sample size, which may limit statistical power and restrict the extent to which the findings can be generalized to the broader patient population in Japan. Further, this study had a relatively short follow-up of 13.8 months, with follow-up currently ongoing. This may potentially impact PFS and OS and explain the differences in treatment response. Additionally, several baseline characteristics were not balanced between the BPd and PVd treatment groups. Despite these imbalances favoring the PVd group, the BPd treatment demonstrated greater overall efficacy compared with PVd. This study was sponsored by GSK and employees of GSK provided input into the design and execution of the study, introducing a potential for sponsor-driven bias. Efforts have been made to minimize the potential for bias, such as the involvement of an IRC in the efficacy assessments.

Belantamab mafodotin is a new treatment option for patients at first relapse or later given its robust efficacy and manageable AEs. This study confirmed the efficacy and safety profile of BPd in Japanese patients with RRMM and ≥ 1 prior therapy, although the small sample size of the study and imbalances in baseline characteristics between the BPd and PVd groups may limit the generalizability of these conclusions. Overall findings were consistent with the global cohort [16]. Although BPd was associated with ocular AEs, they were transient and reversible with dose modifications.

## Supplementary Information

Below is the link to the electronic supplementary material.

Supplementary file1 (DOCX 181 KB)

## Data Availability

Please refer to GSK weblink to access GSK’s data sharing policies and as applicable seek anonymized subject level data via the link https://www.gsk-studyregister.com/en/.

## References

[1] Kumar SK. What is the ideal approach-doublet, triplet, or quadruplet(s)? Hematol Am Soc Hematol Educ Program. 2024;2024:551–60. 10.1182/hematology.2024000581 PMC11665711 39644003

[2] Dimopoulos MA, Moreau P, Terpos E, Mateos MV, Zweegman S, Cook G, et al. Multiple myeloma: EHA-ESMO clinical practice guidelines for diagnosis, treatment and follow-up. Ann Oncol. 2021;32:309–22. 33549387 10.1016/j.annonc.2020.11.014

[3] Iida S, Ishida T, Murakami H, Ozaki S, Abe M, Hata H, et al. JSH practical guidelines for hematological malignancies, 2018: III. Myeloma-1. Multiple myeloma (MM). Int J Hematol. 2019;109:509–38. 30949913 10.1007/s12185-019-02636-8

[4] Sonneveld P, Broijl A. Treatment of relapsed and refractory multiple myeloma. Haematologica. 2016;101:396–406. 27033237 10.3324/haematol.2015.129189 PMC5004403

[5] Mikhael J, Ismaila N, Cheung MC, Costello C, Dhodapkar MV, Kumar S, et al. Treatment of multiple myeloma: ASCO and CCO joint clinical practice guideline. J Clin Oncol. 2019;37:1228–63. 30932732 10.1200/JCO.18.02096

[6] JSH practical guidelines for hematological malignancies, version 3.1. iii. Myeloma; 1. Multiple myeloma (MM). 2024 25 March 2025]; Available from: http://www.jshem.or.jp/gui-hemali/3_1_1.html

[7] Iida S, Yasutomi Y, Samyshkin Y, Chen YC, Chen CC, Lee WS, et al. Characteristics and treatment patterns in patients with multiple myeloma in Japan: a retrospective cohort analysis. PLoS ONE. 2025;20:e0315932. 39847579 10.1371/journal.pone.0315932 PMC11756803

[8] Hartley-Brown MA, Weisel K, Bitetti J, Carter JA, McNamara S, Purser M, et al. Multiple myeloma refractory to lenalidomide: a systematic literature review of trials and real-world evidence. Br J Haematol. 2024;205:780–97. 39031440 10.1111/bjh.19627

[9] Gay F, Zamagni E, Cole CE, Scheid C, Hultcrantz M, Chorazy J, et al. Clinical outcomes associated with anti-CD38-based retreatment in relapsed/refractory multiple myeloma: a systematic literature review. Front Oncol. 2025;15:1550644. 40144211 10.3389/fonc.2025.1550644 PMC11938063

[10] Shah N, Chari A, Scott E, Mezzi K, Usmani SZ. B-cell maturation antigen (BCMA) in multiple myeloma: rationale for targeting and current therapeutic approaches. Leukemia. 2020;34:985–1005. 32055000 10.1038/s41375-020-0734-z PMC7214244

[11] Lassiter G, Bergeron C, Guedry R, Cucarola J, Kaye AM, Cornett EM, et al. Belantamab mafodotin to treat multiple myeloma: a comprehensive review of disease, drug efficacy and side effects. Curr Oncol. 2021;28:640–60. 33494319 10.3390/curroncol28010063 PMC7924384

[12] Tai YT, Mayes PA, Acharya C, Zhong MY, Cea M, Cagnetta A, et al. Novel anti–B-cell maturation antigen antibody-drug conjugate (GSK2857916) selectively induces killing of multiple myeloma. Blood. 2014;123:3128–38. 24569262 10.1182/blood-2013-10-535088 PMC4023420

[13] Morè S, Offidani M, Corvatta L, Petrucci MT, Fazio F. Belantamab mafodotin: from clinical trials data to real-life experiences. Cancers (Basel). 2023;15:2948. 37296910 10.3390/cancers15112948 PMC10251850

[14] Hungria V, Robak P, Hus M, Zherebtsova V, Ward C, Ho PJ, et al. Belantamab mafodotin, bortezomib, and dexamethasone for multiple myeloma. N Engl J Med. 2024;391:393–407. 38828933 10.1056/NEJMoa2405090

[15] Blenrep (belantamab mafodotin) combinations approved by UK MHRA in relapsed/refractory multiple myeloma. 2025 [30 April 2025]; Available from: https://www.gsk.com/en-gb/media/press-releases/blenrep-belantamab-mafodotin-combinations-approved-by-uk-mhra-in-relapsedrefractory-multiple-myeloma/

[16] Dimopoulos MA, Beksac M, Pour L, Delimpasi S, Vorobyev V, Quach H, et al. Belantamab mafodotin, pomalidomide, and dexamethasone in multiple myeloma. N Engl J Med. 2024;391:408–21. 38828951 10.1056/NEJMoa2403407

[17] Blenrep (belantamab mafodotin) combinations approved in Japan for treatment of relapsed/refractory multiple myeloma. 2025 [13 June 2025]; Available from: https://www.gsk.com/en-gb/media/press-releases/blenrep-belantamab-mafodotin-combinations-approved-in-japan/

[18] Blenrep (belantamab mafodotin) combinations approved in EU for treatment of relapsed/refractory multiple myeloma. 2025 [30 July 2025]; Available from: https://www.gsk.com/en-gb/media/press-releases/blenrep-belantamab-mafodotin-combinations-approved-in-eu-for-treatment-of-relapsedrefractory-multiple-myeloma/

[19] Richardson PG, Oriol A, Beksac M, Liberati AM, Galli M, Schjesvold F, et al. Pomalidomide, bortezomib, and dexamethasone for patients with relapsed or refractory multiple myeloma previously treated with lenalidomide (OPTIMISMM): a randomised, open-label, phase 3 trial. Lancet Oncol. 2019;20:781–94. 31097405 10.1016/S1470-2045(19)30152-4

[20] Beksac M, Gonzalez Garcia E, Delimpasi S, Robak P, Karunanithi K, De Arriba F, et al. Belantamab mafodotin plus pomalidomide and dexamethasone vs pomalidomide plus bortezomib and dexamethasone in patients with relapsed/refractory multiple myeloma: a subset analysis in patients who have received 1 prior line of therapy including lenalidomide. Blood. 2024;144:4731.

[21] Raab MS, Zamagni E, Manier S, Rodriguez-Otero P, Schjesvold F, Broijl A. Difficult-to-treat patients with relapsed/refractory multiple myeloma: a review of clinical trial results. EJHaem. 2023;4:1117–31. 38024633 10.1002/jha2.743 PMC10660429

[22] Trudel S, Beksac M, Pour L, Delimpasi S, Quach H, Vorobyev VI, et al. Results from the randomized phase 3 DREAMM-8 study of belantamab mafodotin plus pomalidomide and dexamethasone (BPd) vs pomalidomide plus bortezomib and dexamethasone (PVd) in relapsed/refractory multiple myeloma (RRMM). J Clin Oncol. 2024;42:LBA105.

[23] Yasutomi Y, Ribbands A, Luke E, McNamara S. Treatment landscape and disease burden of patients with multiple myeloma in Japan: a real-world survey. Future Oncol. 2025;21:681–90. 39902484 10.1080/14796694.2025.2460419 PMC11881850

[24] Quach H, Dimopoulos M, Beksac M, Pour L, Delimpasi S, Vorobyev V, et al. Characterization and management of ocular events in patients treated with belantamab mafodotin plus pomalidomide and dexamethasone in the DREAMM-8 study. Clin Lymphoma Myeloma Leuk. 2024;24:S273.

[25] Mateos M-V, Trudel S, Quach H, Robak P, Beksac M, Pour L, et al. Modification of belantamab mafodotin dosing to balance efficacy and tolerability in the DREAMM-7 and DREAMM-8 Trials. Blood Adv. 2025. 10.1182/bloodadvances.2025016949. 40763276 10.1182/bloodadvances.2025016949 PMC12657289

[26] Lonial S, Nooka AK, Thulasi P, Badros AZ, Jeng BH, Callander NS, et al. Management of belantamab mafodotin-associated corneal events in patients with relapsed or refractory multiple myeloma (RRMM). Blood Cancer J. 2021;11:103. 34039952 10.1038/s41408-021-00494-4 PMC8155129

[27] Lu R, Morphey A, Diaz F, Chen J, Razmandi A, Richards T. Management of ocular toxicity in patients receiving Belantamab mafodotin. J Adv Pract Oncol. 2023;14:300–6. 37313276 10.6004/jadpro.2023.14.4.4 PMC10258852

[28] Sunami K, Fujisaki T, Funaki T, Ichii M, Ito S, Matsumoto M, et al. Practical guidance on the clinical management of ocular adverse events associated with belantamab mafodotin in patients with relapsed/refractory multiple myeloma: Recommendations from a Japanese expert panel. Jpn J Clin Oncol. 2025. 10.1093/jjco/hyaf148. 41052095 10.1093/jjco/hyaf148 PMC12675259

