# Supplementary material for: Belantamab mafodotin, pomalidomide, and dexamethasone in Japanese patients with RRMM in the phase 3 DREAMM-8 trial
Source: Int J Hematol. 2026 Jan 6;123(5):686–95. doi: 10.1007/s12185-025-04150-6 (PMC13171770; doi:10.1007/s12185-025-04150-6)
Supplement: Supplementary file 1 — Supplementary file1 (DOCX 181 KB) [file 12185_2025_4150_MOESM1_ESM.docx]

**Supplementary**

**Supplementary material**

**Plain Language Summary (328 words)**

**Why was this study/research done?**

Multiple myeloma is a type of blood cancer that is typically treated with a combination of drugs. However, for most patients these drugs eventually stop working, causing the cancer to worsen or return; this is known as relapsed/refractory multiple myeloma. As a result, there is a need for new treatment options for these patients.

A world-wide clinical study called DREAMM-8 looked at a new drug called belantamab mafodotin (also known as belamaf) in combination with pomalidomide + dexamethasone and compared it to bortezomib with pomalidomide + dexamethasone, which is already being used as standard treatment to treat relapsed/refractory multiple myeloma. The DREAMM-8 study found that the belamaf combination therapy was more effective than the standard treatment at preventing the disease from returning as quickly.

**What did we do?**

We analyzed Japanese patients with relapsed/refractory multiple myeloma who were treated with belamaf (10 patients) or bortezomib (11 patients) with pomalidomide + dexamethasone in the DREAMM-8 study. We looked at how long these patients went without their cancer getting worse or dying and how many patients had reductions to signs and symptoms of cancer with treatment.

**What did we find?**

Patients treated with belamaf combination therapy had a longer period without cancer worsening or death compared with patients treated with bortezomib combination therapy. Also, more patients treated with belamaf combination therapy had reductions to signs and symptoms of cancer compared to patients treated with bortezomib combination therapy.

The side effects reported in the Japanese patients were similar to what was reported in other clinical trials studying belamaf, which included side effects affecting the eye. Patients recovered from these eye side effects when their belamaf treatment was delayed or the dose was reduced. None of the patients had to stop treatment permanently because of these side effects.

**What do these findings mean?**

Japanese patients with relapsed/refractory multiple myeloma treated with the belamaf combination therapy remained stable for a longer time without their cancer worsening compared with the standard treatment.

**Supplementary figures**

**Figure S1.** DREAMM-8 study design

**
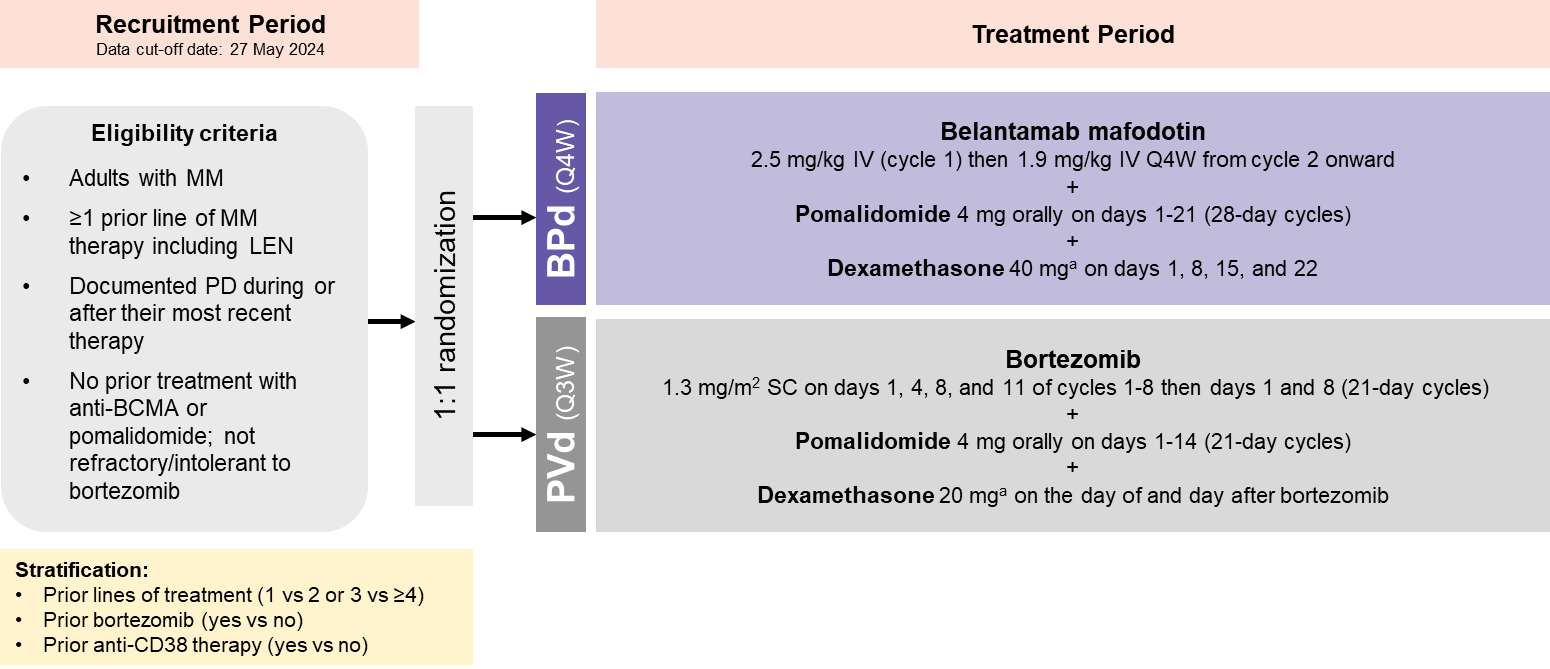
**

^a^ Patients aged >75 years, with comorbidities, or intolerant to 40 mg dose in Arm A or 20 mg dose in Arm B could have dose level reduced to half per investigator discretion.
BCMA, B-cell maturation antigen; BPd, belantamab mafodotin, pomalidomide, and dexamethasone; CD, cluster of differentiation; IV, intravenous; LEN, lenalidomide; MM, multiple myeloma; PD, progressive disease; PVd, pomalidomide, bortezomib, and dexamethasone; Q3W, every 3 weeks; Q4W, every 4 weeks; SC, subcutaneous.

**Figure S2.** Swimmer Plot of Treatment Duration and Response of Each Patient in the BPd Group^a^

**
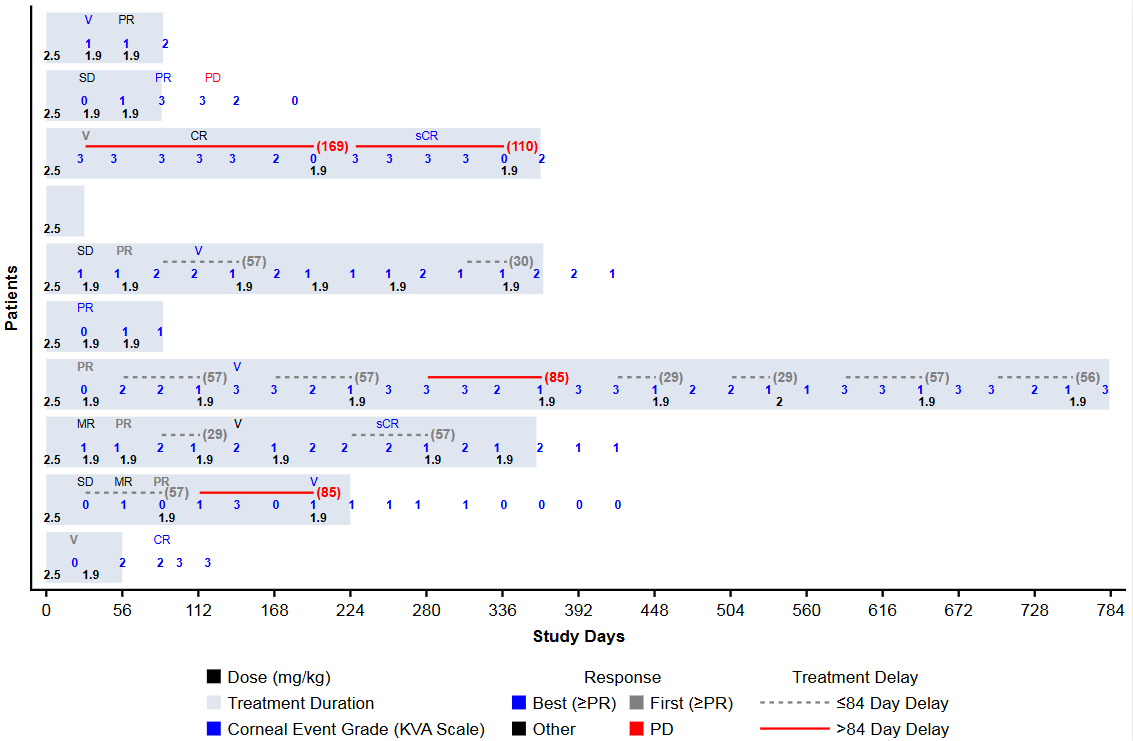
**

^a^ Treatment duration is the time difference between the first dosing date and dosing end date, without accounting for dose interruptions.
CR, complete response; KVA, Keratopathy and Visual Acuity; MR, minimal response; NE, not evaluable; PD, progressive disease; PR, partial response; sCR, stringent complete response; SD, stable disease; V, very good partial response.

**Supplementary tables**

**Table S1.** KVA Scale and Dose Modification Guidelines for Belantamab Mafodotin Treatment-Related Corneal Events Based on KVA Scale

| **KVA Scale** | **Grade 1** | **Grade 2** | **Grade 3** | **Grade 4** |
| --- | --- | --- | --- | --- |
| **Corneal Events** | | | | |
| **Corneal examination findings** | Mild superficial keratopathy^a^ | Moderate superficial keratopathy^b^ | Severe superficial  keratopathy^c^ | Corneal epithelial defect^d^ |
| **Change in Snellen-equivalent BCVA^e,f,g^** | Decline from baseline of 1 line on Snellen-equivalent BCVA | Decline from baseline of 2 or 3 lines (and Snellen-equivalent BCVA not worse than 20/200) | Decline from baseline by more than 3 lines (and Snellen equivalent  BCVA not worse  than 20/200) | Snellen-equivalent BCVA worse  than 20/200 |
| **Recommended dosage modifications per KVA scale** | | | | |
| **Recommended dosage modifications^h^** | Continue treatment at current dose | Withhold treatment and allow to recover to grade ≤1. The patient should restart at a lower dose, as follows:   - If toxicity was identified prior to dosing cycle 2, then the patient should be dosed at 1.9 mg/kg every 4 weeks thereafter, as planned - If toxicity was identified cycle 2+, the patient should be dosed at 1.9 mg/kg every 8 weeks thereafter | Follow the instructions  provided for grade 2 | Hold treatment and allow to recover to grade ≤1. Patient may be restarted following benefit/risk assessment and discussion between  the eye care specialist, the investigator, and  the Sponsor. If restart is approved, participant  should be restarted at 1.4 mg/kg every 8 weeks |

^a^ Mild superficial keratopathy: mild superficial punctate keratopathy (documented worsening from baseline), with or without symptoms. ^b^ Moderate superficial keratopathy: any or a combination of: moderate superficial punctate keratopathy, patchy microcyst-like deposits, subepithelial haze (peripheral), or a new peripheral stromal opacity. ^c^ Severe superficial keratopathy: any or a combination of: severe superficial punctate keratopathy, diffuse microcyst-like deposits involving the central cornea, subepithelial haze (central), or a new central stromal opacity. ^d^ Corneal epithelial defects such as corneal ulcers. Corneal ulcer by definition means an epithelial defect with underlying stromal infiltration. ^e^ Changes in visual acuity due to treatment-related corneal findings apply: 1. For patients who have BCVA worse than 20/20 in either eye at baseline, dose modification for that eye will be determined by the worsening of vision from baseline only (not by absolute BCVA at the visits). 2. If a patient has a baseline BCVA of 20/200 or worse in an eye, then belantamab mafodotin related changes in vision in the other eye will drive the dose modification. If a patient has baseline BCVA of 20/200 or worse in both eyes, then the decision to delay or reduce belantamab mafodotin dose will be based on the Principal Investigator’s assessment of benefit vs risk based on corneal examination findings following a discussion with a qualified eye care specialist such as an ophthalmologist/optometrist. 3. Dose modification should be based on the most severe grade. If eyes differ in severity, the dose modification guideline should be applied based on the more severe eye. ^f^ Snellen equivalent BCVA is recommended to be tested on a visual acuity chart which has an approximately equal number of letters per line and equal spacing between lines. ^g^ If a patient has cataract surgery during the study, the BCVA should be re-baselined (after the BCVA stabilizes if there are no corneal findings but prior to any further belantamab mafodotin administration) and subsequent visual acuity must then be assessed from this new “best” baseline value. ^h^ Dose modification should be based on the most severe finding. If eyes differ in severity, the dose modification guideline should be applied based on the more severe eye. Dose reductions of belantamab mafodotin will be triggered by grade 2 or worse events of ocular exam findings or decrease in BCVA on day of dosing.
BCVA, Best-Corrected Visual Acuity; KVA, Keratopathy Visual Acuity.

**Table S2.** Patients With CR or Better Who Achieved MRD negative Status in Either Treatment Group

|  | BPd (N=10) | PVd (N=11) |
| --- | --- | --- |
| MRD-negative status^a^ | | |
| Patients with complete response or better, n (%) | 2 (20.0) | 2 (18.2) |
| 95% CI | 2.5–55.6 | 2.3–51.8 |
| Patients with very good partial response or better, n (%) | 4 (40.0) | 2 (18.2) |
| 95% CI | 12.2–73.8 | 2.3–51.8 |
| MRD-negative status sustained for ≥12 months | | |
| Patients with complete response or better, n (%) | 0 | 1 (9.1) |
| 95% CI | 0.0–30.8 | 0.2–41.3 |

^a^ MRD-negative status was assessed by means of next-generation sequencing at a sensitivity of 10^−5^ or lower.
BPd, belantamab mafodotin, pomalidomide, and dexamethasone; CI, confidence interval; CR, complete response; MRD, minimal residual disease; PVd, pomalidomide, bortezomib, and dexamethasone.

**Table S3.** Adverse Events Reported in Either Treatment Group (Safety Population)

|  | BPd (N=10) | PVd (N=11) |
| --- | --- | --- |
| Any AE, n (%) | 10 (100) | 11 (100) |
| AEs related to any study treatment^a^ | 9 (90) | 11 (100) |
| Grade 3/4 AEs | 9 (90) | 9 (82) |
| Grade 3/4 AEs related to any study treatment^a^ | 8 (80) | 7 (64) |
| AEs leading to permanent discontinuation of any study treatment | 1 (10) | 4 (36) |
| AEs related to any study treatment and leading to permanent of any study treatment^a^ | 0 | 3 (27) |
| AEs leading to dose reduction | 6 (60) | 5 (45) |
| AEs leading to dose interruption/delay | 7 (70) | 9 (82) |
| Any SAE, n (%) | 5 (50) | 4 (36) |
| SAEs related to any study treatment^a^ | 2 (20) | 1 (9) |
| Fatal SAEs | 1 (10) | 1 (9) |
| Fatal SAEs related to any study treatment^a^ | 0 | 0 |

^a^ Related to any study treatment' included responses of 'Yes' and missing responses to the following question: "Is there a reasonable possibility that the AE may have been caused by the study treatment?".
AE, adverse event; BPd, belantamab mafodotin, pomalidomide, and dexamethasone; PVd, pomalidomide, bortezomib, and dexamethasone; SAE, serious adverse event.

**Table S4.** Adverse Events by SOC in ≥20% of Patients in Either Treatment Group (Safety Population)

| SOC, n (%) | BPd (N=10) | PVd (N=11) |
| --- | --- | --- |
| Any event | 10 (100) | 11 (100) |
| Investigations | 9 (90) | 7 (64) |
| Eye disorders | 9 (90) | 3 (27) |
| Gastrointestinal disorders | 7 (70) | 8 (73) |
| Infections and infestations | 7 (70) | 4 (36) |
| Skin and subcutaneous tissue disorders | 6 (60) | 5 (45) |
| General disorders and administration site conditions | 5 (50) | 7 (64) |
| Nervous system disorders | 5 (50) | 4 (36) |
| Blood and lymphatic system disorders | 4 (40) | 7 (64) |
| Metabolism and nutrition disorders | 3 (30) | 4 (36) |
| Immune system disorders | 3 (30) | 0 |
| Musculoskeletal and connective tissue disorders | 2 (20) | 6 (55) |
| Renal and urinary disorders | 2 (20) | 2 (18) |
| Injury, poisoning and procedural complications | 2 (20) | 1 (9) |
| Respiratory, thoracic and mediastinal disorders | 1 (10) | 3 (27) |
| Vascular disorders | 1 (10) | 3 (27) |

BPd, belantamab mafodotin, pomalidomide, and dexamethasone; PVd, pomalidomide, bortezomib, and dexamethasone; SOC, system organ class.
